# Omentum preservation versus complete omentectomy in gastrectomy for gastric cancer (OMEGA trial): study protocol for a randomized controlled trial

**DOI:** 10.1186/s13063-024-08396-z

**Published:** 2024-09-04

**Authors:** K. Keywani, W. J. Eshuis, A. B. J. Borgstein, M. J. van Det, P. van Duijvendijk, B. van Etten, P. P. Grimminger, J. Heisterkamp, S. M. Lagarde, M. D. P. Luyer, S. R. Markar, S. L. Meijer, J. P. E. N. Pierie, F. Roviello, J. P. Ruurda, J. W. van Sandick, M. Sosef, B. P. L. Witteman, W. O. de Steur, B. I. Lissenberg-Witte, M. I. van Berge Henegouwen, S. S. Gisbertz

**Affiliations:** 1 Amsterdam UMC Location, Department of Surgery, Amsterdam, the Netherlands; 2 Cancer Center Amsterdam, Cancer Treatment and Quality of Life, Amsterdam, the Netherlands; 3 Ziekenhuis Groep Twente, Department of Surgery, Almelo, the Netherlands; 4 Gelre Ziekenhuis, Department of Surgery, Apeldoorn, the Netherlands; 5 Department of Surgery, Universitair Medisch Centrum Groningen, Groningen, the Netherlands; 6 Department of General, Visceral and Transplant Surgery, University Medical Center of the Johannes Gutenberg University, Mainz, Germany; 7 Department of Surgery, Elisabeth Tweesteden Ziekenhuis, Tilburg, the Netherlands; 8 Department of Surgery, Erasmus Medisch Centrum, Rotterdam, the Netherlands; 9 Department of Surgery, Catharina Ziekenhuis, Eindhoven, the Netherlands; 10 Nuffield Department of Surgery, University of Oxford, Oxford, UK; 11 Amsterdam UMC location University of Amsterdam, Department of Pathology, Amsterdam, the Netherlands; 12 Department of Surgery, Medisch Centrum Leeuwarden, Leeuwarden, the Netherlands; 13 Department of Surgery, Azienda Ospedaliera Universitaria, Siena, Italy; 14 Universitair Medisch Centrum Utrecht, Department of Surgery, Utrecht, the Netherlands; 15 The Netherlands Cancer Institute - Antoni van Leeuwenhoek, Department of Surgery, Amsterdam, the Netherlands; 16 Department of Surgery, Zuyderland ziekenhuis, Heerlen, the Netherlands; 17 Department of Surgert, Rijnstate Ziekenhuis, Arnhem, the Netherlands; 18 Department of Surgery, Leids Universitair Medisch Centrum, Leiden, the Netherlands; 19 Department of Epidemiology and Data Science, VU University Amsterdam, Amsterdam, the Netherlands

**Keywords:** Gastric cancer, Omentectomy, Survival, Randomized controlled trial

## Abstract

**Background:**

Potentially curative therapy for locally advanced gastric cancer consists of gastrectomy, usually in combination with perioperative chemotherapy. An oncological resection includes a radical (R0) gastrectomy and modified D2 lymphadenectomy; generally, a total omentectomy is also performed, to ensure the removal of possible microscopic disease. However, the omentum functions as a regulator of regional immune responses to prevent infections and prevents adhesions which could lead to bowel obstructions. Evidence supporting a survival benefit of routine complete omentectomy during gastrectomy is lacking.

**Methods:**

OMEGA is a randomized controlled, open, parallel, non-inferiority, multicenter trial. Eligible patients are operable (ASA < 4) and have resectable (≦ cT4aN3bM0) primary gastric cancer. Patients will be 1:1 randomized between (sub)total gastrectomy with omentum preservation distal of the gastroepiploic vessels versus complete omentectomy. For a power of 80%, the target sample size is 654 patients. The primary objective is to investigate whether omentum preservation in gastrectomy for cancer is non-inferior to complete omentectomy in terms of 3-year overall survival. Secondary endpoints include intra- and postoperative outcomes, such as blood loss, operative time, hospital stay, readmission rate, quality of life, disease-free survival, and cost-effectiveness.

**Discussion:**

The OMEGA trial investigates if omentum preservation during gastrectomy for gastric cancer is non-inferior to complete omentectomy in terms of 3-year overall survival, with non-inferiority being determined based on results from both the intention-to-treat and the per-protocol analyses. The OMEGA trial will elucidate whether routine complete omentectomy could be omitted, potentially reducing overtreatment.

**Trial registration:**

ClinicalTrials.gov NCT05180864. Registered on 6^th^ January 2022.

**Supplementary Information:**

The online version contains supplementary material available at 10.1186/s13063-024-08396-z.

## Background

Gastric cancer is the fifth most prevalent type of cancer worldwide and the third most common cause of cancer-related death [1]. Overall, survival has improved in recent decades with the introduction of (neo)adjuvant therapy; as yet, a radical gastrectomy remains the foundation of curative treatment for advanced gastric cancer. An oncological resection involves a radical (R0) gastrectomy with a modified D2 lymphadenectomy. Generally, a complete resection of the greater omentum is also performed to ensure the removal of possible micrometastatic disease.

The omentum contributes to the defense against infections, by functioning as a regulator of regional immune responses [2–5]. Furthermore, the omentum prevents the occurrence of adhesions that can lead to small bowel obstruction [6]. In (laparoscopic) gastric cancer surgery, omentectomy can be a time-consuming and technically demanding procedure which has been shown to increase the risk of intraoperative injuries to the colon and mesocolon [7]. Additionally, while rare cases of omental infarction have been reported following omentum preservation [8], complete omentectomy has been associated with an increased risk of postoperative complications, such as abdominal abscesses, wound infections, and ileus because of mechanical small bowel obstruction following various types of surgery [7, 9–12].

Evidence supporting routine complete omentectomy in gastrectomy for cancer is currently lacking. Recently, the short-term results of a phase II trial were published [13]. No difference was found in postoperative morbidity between patients who underwent a complete omentectomy versus partial omentectomy during radical gastrectomy. Several non-randomized studies have shown no survival difference between partial and complete omentectomy as part of radical gastrectomy for gastric cancer [14–17]. These studies suggest that a total omentectomy can be omitted as part of potential curative surgery. However, most of these studies were performed in Asian countries, where gastric cancer is more prevalent and more patients are diagnosed with early gastric cancer as a consequence of screening. In contrast to Western practices, patients seldom receive perioperative chemotherapy, and an open gastrectomy is more prevalent as a surgical approach. Hence, a comparison of Asian and Western studies on gastric cancer should be made with caution.

To date, the influence of omentectomy on survival has not yet been investigated in a randomized controlled trial in a Western gastric cancer population. Some prospective studies were performed in which the omentum in all patients was completely resected and separately pathologically investigated [18, 19]. One study showed that the incidence of metastases in the greater omentum was 5%, and when present, was associated with advanced disease and a non-radical resection in all these patients [18]. With a median survival of 7 months, none survived more than 2 years [20]. Another study reported that in four (8%) patients the greater omentum harbored tumor deposits, of whom all experienced recurrent disease within 1 year after surgery, and lymph node metastases were found in the omentum in one (2%) patient, who was disease-free after 20 months, although the exact location of this lymph node metastasis was not known [19]. A high-quality trial is needed to evaluate the non-inferiority of omentum-preserving gastrectomy in a Western population. In this randomized controlled trial, we test our hypothesis that omentum preservation during gastrectomy in patients with locally advanced gastric cancer is non-inferior to complete omentectomy in terms of 3-year overall survival.

## Methods

This protocol was developed according to the Standard Protocol Items: Recommendations for Interventional Trials (SPIRIT 2013 Checklist) (Additional file 1: S1) [21].

### Objective

The primary objective is to compare the 3-year overall survival after omentum preservation versus complete omentectomy in gastric cancer patients undergoing potentially curative resection for locally advanced disease.

### Study design and setting

The OMEGA study is a randomized controlled, open, parallel, non-inferiority, multicenter trial. Eligible patients have to be operable (ASA < 4) with resectable (≦ cT4aN3bM0) gastric cancer. Patients will be 1:1 randomized between radical (sub)total gastrectomy with omentum preservation versus complete omentectomy. Patients will be stratified according to center, neoadjuvant therapy, and type of surgery (total or subtotal gastrectomy). In total, 654 patients will be randomized. The study will be conducted in five Dutch university hospitals, eight Dutch teaching hospitals, and three international university hospitals (Siena, Oxford, and Mainz), all performing more than 20 gastrectomies annually.

### Inclusion criteria

In order to be eligible to participate in this study, a subject must meet all of the following criteria:

- Primary resectable gastric adenocarcinoma, clinical stage T1-4aN0-3M0
- ASA 1–3 (able to undergo surgery)
- Scheduled for open or minimally invasive (sub)total gastrectomy with modified D2-lymphadenectomy, with or without perioperative chemotherapy
- Age above 18
- Able to complete questionnaires in Dutch, English, or Italian
- Written informed consent
- Esophageal invasion < 2 cm defined from the upper margin of the gastric rugae as determined by endoscopy

### Exclusion criteria

A potential subject who meets any of the following criteria will be excluded from participation in this study:

- Gastric cancer clinically staged as T1N0
- Locally advanced gastric cancer requiring multi-visceral resection
- Pregnancy
- Previous malignancy (excluding non-melanoma skin cancer, pancreatic neuroendocrine tumor (pNET) < 2 cm, and gastrointestinal stromal tumor (GIST) < 2 cm), unless no evidence of disease and diagnosed more than 3 years before diagnosis of gastric cancer, or with a life expectancy of more than 5 years from date of inclusion
- Serious concomitant systemic disorders that would compromise the safety of the patient or his/her ability to complete the study, at the discretion of the investigator
- Previous gastric or omental surgery, with the exclusion of a gastric perforation
- Indication for thoracotomy/thoracoscopy

### Recruitment and participants

**Fig. 1:**
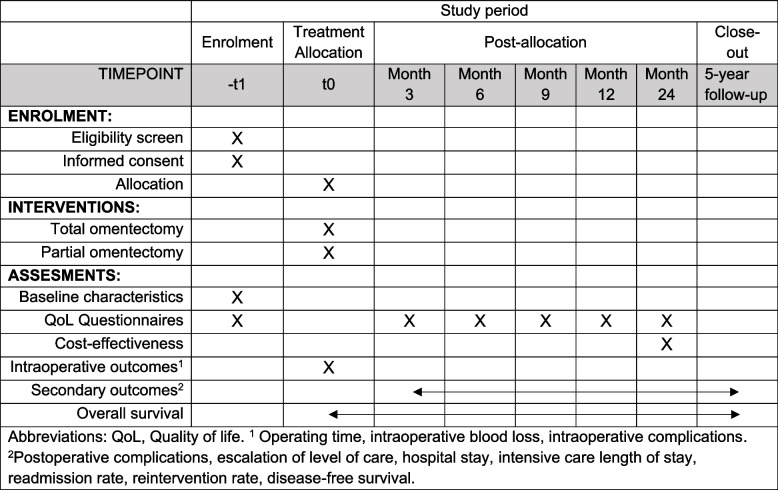
The schedule of enrollment, interventions, and assessment according to SPIRIT

Eligible patients will be approached for entry into the trial during an outpatient visit at the surgery department after the diagnosis of gastric cancer. The treating surgeon will perform the eligibility screening and ask the patient for consent to be contacted by a local researcher regarding medical scientific research. The rationale for the study is explained to the patient by a local researcher. A written patient information sheet is provided and patients will be given the opportunity to ask questions. After a sufficient reflection period (minimum of 1 week), the willing patients are asked to sign the informed consent before any study intervention. The SPIRIT schedule of enrollment, interventions, and assessment is shown in Fig. 1 [21]. Written informed consent is obtained by a local researcher. When consent has been obtained, the original form is kept in the study file and a copy is given to the patient. Patient’s data will be uploaded into the database using codes.

### Sample size

The primary endpoint is 3-year overall survival. According to survival rates from the Dutch Cancer Registry (NKR), 3-year overall survival after gastrectomy is approximately 50% in the Netherlands. Under the common assumption of exponential survival times, a hazard ratio of 0.862 under the alternative hypothesis, at least 50% and 45% expected events (i.e., death) in the control arm and experimental arm, respectively, at the minimum follow-up of 3 years, 298 events are needed in total to achieve 80% power at a one-sided significance level of 5% with a non-inferiority hazard ratio of 1.15 (PASS 15 Power Analysis and Sample Size Software (2017). NCSS, LLC. Kaysville, Utah, USA, ncss.com/software/pass), resulting in 314 patients per study arm. Dropouts will be rare (mostly due to loss to follow-up, which is quite rare in cancer patients), with proportion dropping out expected to be at most 5%. After correction for drop-out, we plan to include 327 patients in each of the two arms (654 in total).

### Randomization and blinding

**Fig. 2:**
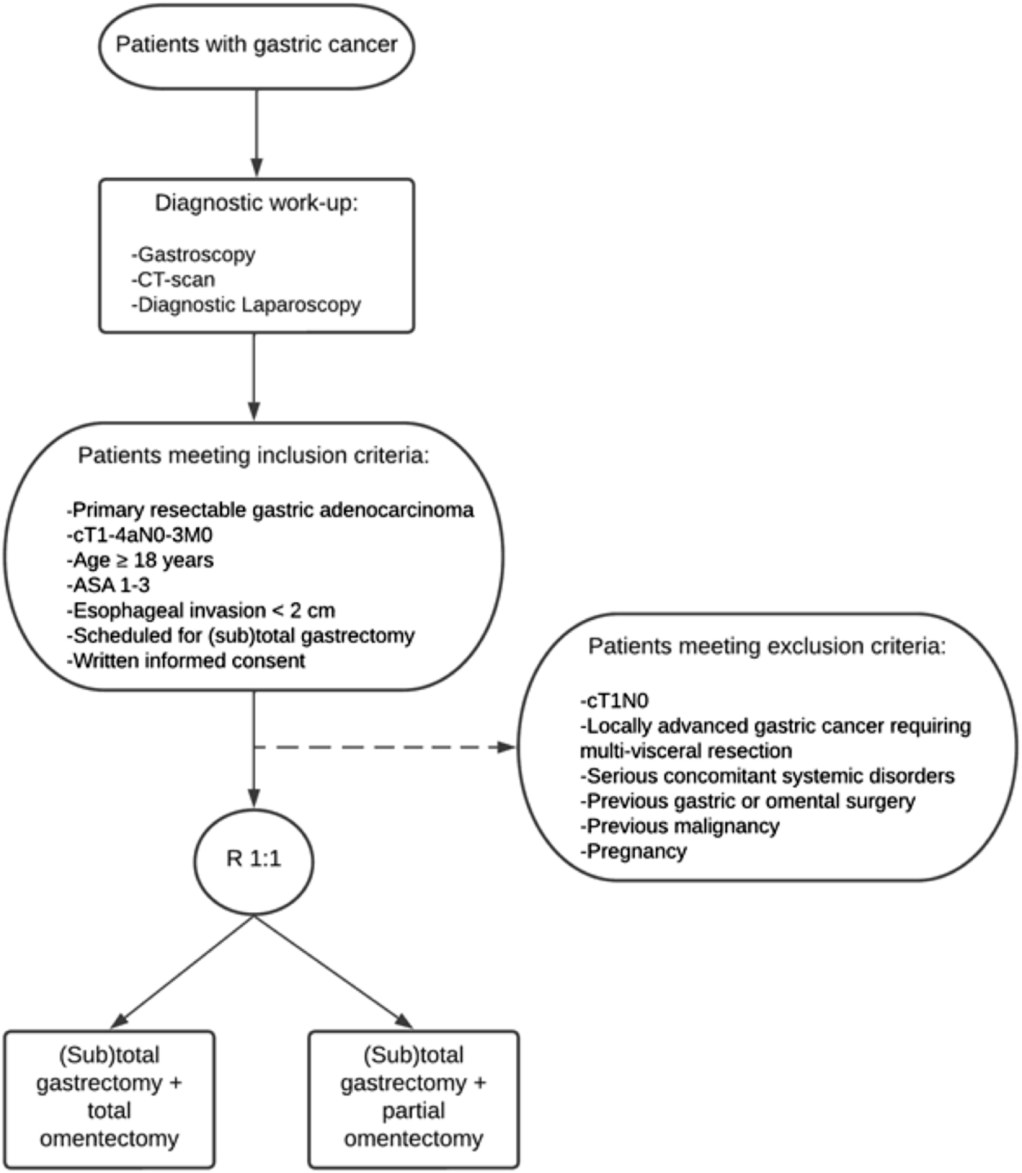
Flow diagram study participation

Patients will be randomized in a 1:1 ratio between gastrectomy with complete omentectomy or omentum preservation (Fig. 2). Randomization will be performed preoperatively by the central coordinating researcher, after informed consent has been obtained by a local researcher. Randomization will be done with the use of an online computer program, with varying blocks, and stratified by participating center, neoadjuvant therapy, and type of operation (subtotal or total gastrectomy). Blinding for the type of investigational treatment will not be performed. Patients will be excluded and replaced with new subjects if metastases and/or non-resectability are detected during surgery. As a consequence, the protocol treatment will also be terminated when omental metastatic disease is detected during surgery and these patients will be replaced with new subjects.

### Treatment

Patients will be staged according to the AJCC 8^th^ edition of gastric cancer staging [22]. Staging will be done according to international gastric cancer guidelines [23, 24]. Patients will be diagnosed by upper GI endoscopy with biopsies, and staged with CT scanning of the thorax and abdomen. In addition, staging laparoscopy will be performed in patients with ≥ cT3 stage/cN + tumors with peritoneal lavage for cytological examination (optional). Endoscopic ultrasound will be performed on indication. Patients will usually be treated with perioperative chemotherapy according to the FLOT scheme [25], unless contraindicated because of patient factors (age, comorbidities) or tumor factors (bleeding or obstruction).

In patients scheduled for perioperative chemotherapy, the gastrectomy will be performed approximately 4 – 8 weeks after completion of the neoadjuvant phase. Those not treated with chemotherapy will be directly scheduled for surgery. Surgery will be performed according to treatment allocation: omentum preservation or complete omentectomy.

### Gastrectomy with omentectomy

The operation will start with the establishment of resectable disease. Then, the abdominal cavity will be washed with saline 0,9% at body temperature (optional). After 2 min, at least 500 mL saline will be collected and sent in for pathological examination. In case of established resectable disease, a laparoscopic or open radical (sub)total gastrectomy will be performed, as both procedures have similar oncological results in RCTs [26, 27]. A subtotal gastrectomy may be performed if a tumor-free proximal margin of at least 6 cm can be obtained. If this cannot be achieved, a frozen section should be performed to ensure a negative proximal margin. If a distal resection margin of less than 6 cm is obtained, and a more extended resection is possible, a frozen section is advised according to the international guidelines [23, 24]. In all other cases, a total gastrectomy should be performed.

**Fig. 3:**
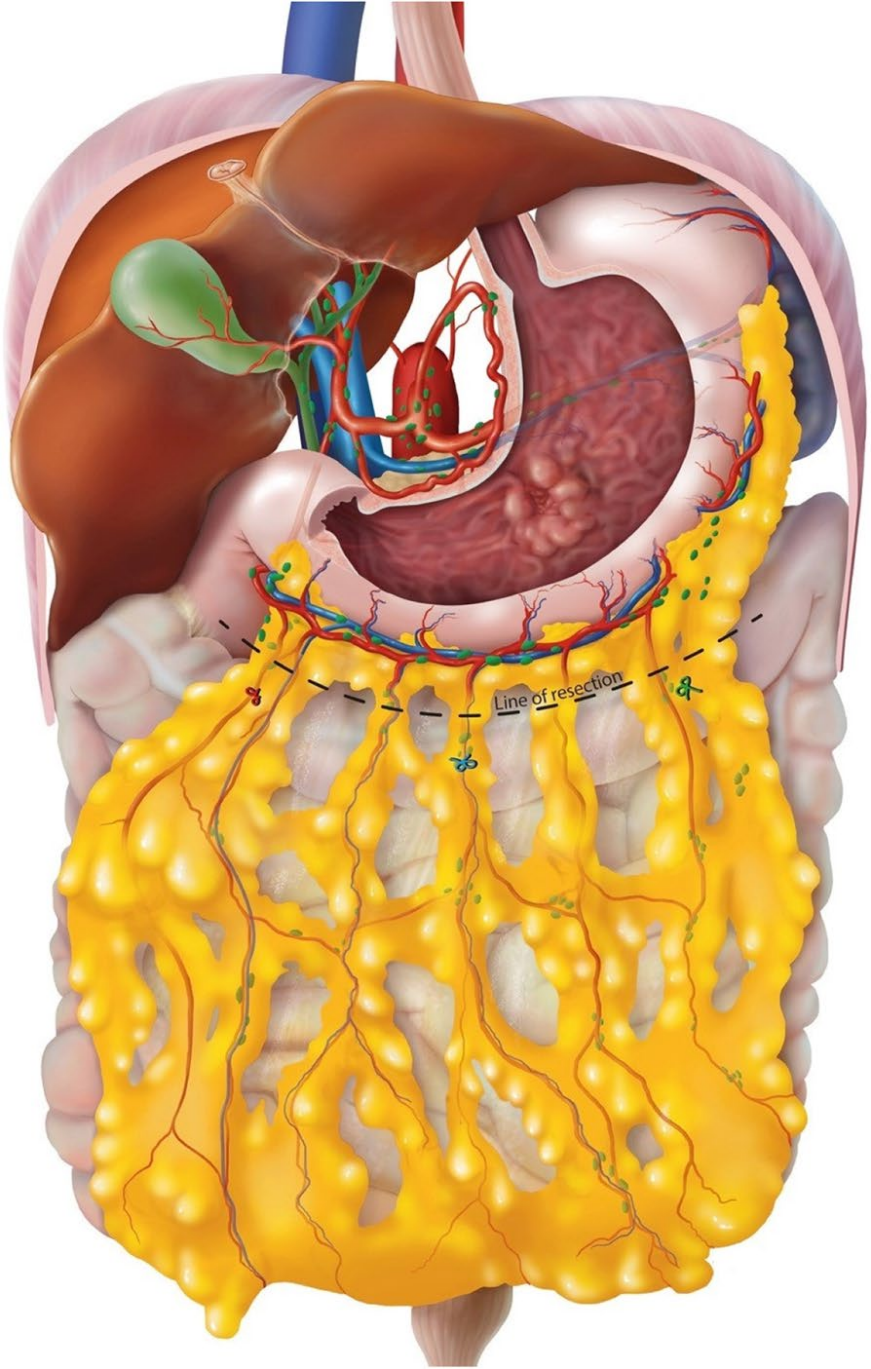
Line of resection and anatomic markings. Red suture: hepatic marking. Blue suture: cranial marking, in the middle of the omentum. Green suture: spleen marking. Dotted line: line of resection distal to the gastroepiploic arcade

The hepatogastric ligament is opened close to the liver. The right and left gastric artery and vein are ligated at the base. A modified D2 lymphadenectomy (according to the Japanese gastric cancer guideline 6^th^ edition) is performed: stations 1, 3–8a, 9, 11p, and 12a in subtotal gastrectomy and stations 1–7, 8a, 9, 11p, 11d, and 12a in total gastrectomy [24]. A complete omentectomy is performed en bloc or separately (by discretion of the surgeon). The right and left gastroepiploic artery and vein are ligated and the short gastric vessels in case of a total gastrectomy. The duodenum and esophagus or proximal stomach are divided and the specimen is removed. After resection, a Roux-en-Y or Billroth II reconstruction (esophagojejunostomy or gastrojejunostomy) will be performed. A Roux-Y entero-enterostomy is created with a biliary limb of approximately 20 cm and an alimentary limb of approximately 50 cm. In case of Billroth II reconstruction, a loop of jejunum 10 to 15 cm distal to the duodenojejunal flexure is brought up to the remnant stomach to create a gastrojejunostomy. The D1 + and D2 lymph node stations and station 7 are sent in separately to the pathology department. The perigastric lymph nodes (D1 nodes except station 7) are marked on the specimen with sutures or beads. In case of a complete omentectomy the omentum is ex-vivo or in-vivo dissected of the specimen, marked (Fig. 3), and send in separately for pathological examination. The specimen will be marked cranially, and at both the liver and spleen sites.

### Gastrectomy with omentum preservation

The same procedure is performed with regard to gastrectomy and lymphadenectomy. In this group, the gastrocolic ligament is divided 3 cm distal to the gastroepiploic vessels, and the omentum proximal of the level of the attachment of the omentum on the transverse colon is left in situ (Fig. 3). If possible, some branches to the omentum of the left gastro-epiploic vessels may be preserved. Assessment of perfusion of the remaining omentum will be performed (optional) using indocyanine green (ICG) and a near-infrared camera (NIR). ICG 0.1 mg/kg will be administered through a peripheral infusion cannula on the right or left arm after which the cannula is flushed. A maximum overview of the preserved omentum is created by positioning the fluorescence camera above the omentum (using the left or right trocar of the surgeon in laparoscopic surgery). Time to first fluorescence signal and time to complete enhancement of the omentum will be recorded as well as whether areas of the omentum remain non-fluorescent, which will be registered as estimated number of cm^2^ and percentage of total omentum. In case of non-enhancement of (parts of) the omentum by ICG, the omentum will be left in situ. However, the final decision whether or not to remove the omentum is left at the discretion of the surgeon. Any additional resection of the omentum is registered in the CRF.

### Postoperative management

Patients in both study groups will receive similar standard postoperative treatment. In all centers, an ERAS protocol has been implemented [28].

### Pathology

The gastrectomy specimen and lymphadenectomy specimens will be processed and analyzed according to national and international guidelines by the Department of Pathology [29, 30]. The total number and localization of excised lymph nodes in the greater omentum will be determined, as well as the number of tumor-involved nodes or tumor deposits (where no lymph nodes are recognized). The greater omentum will be divided into three areas according to the anatomical marks provided by the surgeon (spleen, cranial/middle, liver markings: Fig. 3). All lymph nodes under 5 mm will be totally embedded for microscopic evaluation; larger lymph nodes will be sliced into Sects. 3–4 mm thick and then totally embedded. Microscopically, a circumscript area of lymphoid cells containing a follicular architecture and/or a subcapsular sinus is identified as a lymph node. In case of tumor deposits or areas suspicious for tumor deposits, the largest diameter is provided and one single slice is embedded. The embedded material will be processed according to routine procedures. One H&E stained slide will be studied for every paraffin block. In case the lymph nodes from the omentum are not macroscopically or histologically (H&E) tumor positive or extensive response to neoadjuvant therapy is observed additional keratin stains will be performed.

### Surgical quality assurance (SQA)

To be credentialed for trial entry, the institute must perform at least twenty gastrectomies annually. Furthermore, the performing surgeon has to have performed a minimum of 50 gastrectomies in total, and watch a provided sample video of gastrectomy with and without total omentectomy. A non-edited video of both procedures is then recorded during the procedure by the performing surgeon for assessment by the SQA team. The SQA team assesses the surgical quality using a standardized form (Additional file 1: S2). During the trial, every 15^th^ inclusion from each study site in the trial, a video of the surgical procedure will be recorded and assessed by the SQA team (Additional file 1: S3). The SQA team consists of two surgeons from the project group.

### Primary endpoint

The primary endpoint is overall survival at 3 years after surgery, defined as the period of time between operation and death from any cause. Patients alive at the last follow-up will be censored*.*

### Secondary endpoints

Secondary outcomes are operating time, intraoperative blood loss, intraoperative complications, postoperative complications, defined [31] and graded according to the Clavien-Dindo classification [32] and comprehensive complication index (CCI) [33] late intra-abdominal complications, defined as complications related to the initial operation, occurring between > 30 days and 5 years after surgery, (y)pTNM status, the location and presence of metastates in the omentum in the total omentectomy group, distribution of lymph node metastases, R0 resection rate, defined as the percentage of patients that underwent a microscopically complete (R0) resection, rate of malignant cells in cytology (optional), serum CRP levels at postoperative days 3, 5, and 7 (optional), molecular subclassification of gastric cancer (optional), ICG fluorescent enhancement of omentum in omentum preservation group (optional), compliance to allocated treatment (including the actual number of patients who underwent either omentum preservation or complete omentectomy), escalation of level of care, hospital stay, defined as time interval between date of surgery and date of hospital discharge, intensive care length of stay, readmission rate within 30 days after surgery, reintervention rate within 30 days after surgery, reoperation rate within 3 years after surgery, cost effectiveness, quality of life at baseline, 3, 6, 9, 12, and 24 months (with the following questionnaires collected in collaboration with the POCOP study: EQ-5D-5L, QLQ-C30, QLQ-OG25) [34], the occurrence of peritoneal metastases during follow-up and 3- and 5-year disease-free survival, defined as the period of time from operation to locoregional recurrence, distant metastases, recurrent gastric cancer or death from any cause, 5-year overall survival, defined as the period of time from operation to death from any cause. Patients alive and free of all these events will be censored at the last follow-up.

### Baseline characteristics

Baseline characteristics will include age, sex, medical history, previous surgery, BMI, weight loss, American Society of Anaesthesiologists class (ASA), WHO performance status, Charlson Comorbidity Index, tumor location and differentiation, Lauren classification, cTNM stage, and regimen and completion of perioperative therapy according to type.

### Follow-up

Follow-up visits (telephone, video, or visit) will be scheduled 2 weeks after surgery, followed by every 3 months for the first year, every 6 months the second to fourth year, and once yearly until the fifth postoperative year. Patients will be followed up with additional diagnostics (CT thorax/abdomen, endoscopy, EUS) on indication only, according to the international gastric cancer guidelines [23, 24].

### Safety and Data Safety Monitoring Board (DSMB)

All adverse events considered related to the experimental intervention reported spontaneously by the subject or observed by the investigator or his staff occurring until discharge from the hospital will be recorded. Serious adverse events will be reported through a web portal (www.toetsingonline.nl) to the Dutch central committee on research involving human subjects and the institutional review board (Medical Ethics Committee of Amsterdam UMC). A single formal interim analysis will take place after 145 deaths (approximately 50% of the total number expected during the trial period) have been observed. At this interim analysis, the trial will be stopped for futility if the hazard ratio exceeds the non-inferiority hazard ratio of 1.15. The trial will be stopped for superiority if the *p*-value for testing HR = 1 versus HR < 1 is below 0.001 (Peto approach). Stopping and declaring the experimental treatment non-inferior will not be considered as this is generally not recommended. The DSMB will be informed about the details and outcome of this analysis. The DSMB may advise to terminate the trial prematurely in case of clear evidence of harm of partial omentectomy or external evidence, such as other trials or published data, not available during the start of this trial. The advice(s) of the DSMB will only be sent to the sponsor of the study. Should the sponsor decide not to fully implement the advice of the DSMB, the sponsor will send the advice to the reviewing METC, including a note to substantiate why (part of) the advice of the DSMB will not be followed. The DSMB will consist of the following independent members: an epidemiologist/statistician, a surgeon, and a medical oncologist. The DSMB committee meetings will be held according to the following schedule: before enrolment of study participants, yearly until the last follow-up (after inclusion of the first study participant), and after 145 deaths.

### Site monitoring and data processing

The study will be considered medium risk according and will be monitored according to The Netherlands Federation of University Medical Centres guidelines. Site monitoring will be performed by an independent Clinical Research Monitor of the Amsterdam UMC. Collected data are treated confidentially and pseudonymized. Patients will be coded by a numeric randomization code, and the (local) principal investigator will be the only one with access to it. The source data will be accessible by the principal investigator only and stored digitally for 15 years after the last patient’s follow-up is completed.

### Ethics and dissemination of trial findings

The OMEGA trial will be conducted according to the principles of the Declaration of Helsinki (64th WMA General Assembly, Fortaleza, Brazil, October 2013), “good clinical practice” guidelines, and in accordance with the local laws and regulations, such as in the Netherlands the Medical Research Involving Human Subjects Act (WMO). The independent ethics review board of the Amsterdam UMC (Amsterdam, the Netherlands; (NL80328.029.22)) has approved the study protocol. Furthermore, the IRBs of the participating centers will need to approve local feasibility before enrolling patients in the trial. All protocol amendments will be notified to the METC and after approval updated on ClinicalTrials.gov. This trial was registered on 6^th^ January 2022, in the ClinicalTrials.gov register under identification number NCT05180864.

The results of this trial will be submitted for publication in a peer-reviewed scientific journal regardless of the outcomes, even if the results contradict the hypotheses. Authorship will be based on the International Committee of Medical Journal Editors criteria for authorship.

### Statistical analysis

Descriptive statistics will be calculated to summarize patients’ groups included in each trial arm. Mean and standard deviation will be presented for normally distributed continuous variables. Median plus interquartile range (IQR) will be presented for continuous variables that are skewed and for ordinal variables. Dichotomous and nominal data will be summarized by means of frequencies and percentages.

Non-inferiority of the experimental treatment in terms of overall survival will be tested using Cox regression. Non-inferiority will be concluded if the upper limit of the 90% confidence interval falls below the non-inferiority hazard ratio of 1.15, corresponding to a one-sided non-inferiority test at a significance level of 5%. Survival will be presented graphically using Kaplan–Meier curves. All analyses will be according to the intention-to-treat principle. In addition, a per-protocol analysis will also be performed for the primary outcome. The experimental treatment will be declared non-inferior if non-inferiority is shown in both the intention-to-treat and the per-protocol analysis.

Secondary outcomes will be compared between groups using appropriate statistical methods, independent samples *t*-test for normally distributed continuous outcomes, and Mann–Whitney *U* tests for continuous outcomes that are not normally distributed or ordinal outcomes. Categorical outcomes will be compared using the chi-square test or Fisher’s exact test in case of low (expected) cell counts. Repeatedly measured outcomes will be compared between arms using linear mixed models. Secondary time-to-event outcomes will be compared using the log-rank test. Secondary endpoints will be tested at a two-sided significance level of 5%. Effect sizes suitable for the type of outcome measure will be provided (mean differences, ratio of geometric means, relative risks, hazard ratios) together with their 95% confidence interval. The Holm procedure will be used to correct for multiple testing.

Subgroup analysis for the effect of experimental treatment on overall survival will be performed for the following subgroups: patient characteristics (age, gender), diffuse/intestinal type gastric tumor, subtotal/total gastrectomy, and minimally invasive/open gastrectomy. Effect modification will use Cox regression with the subgroup variable, the arm, and their two-way interaction. Additionally, stratified analyses will be performed where HR is calculated separately in each of the subgroups.

Quality of life data will be graphically represented across all time points and analyzed according to the manuals and will presented as domain and summarized scores. Questionnaire outcome comparisons will be analyzed using linear mixed models.

## Discussion

The OMEGA trial is an international randomized controlled trial, that will investigate whether omentum preservation during gastrectomy for cancer is non-inferior to the current practice of complete omentectomy in terms of overall survival. All published studies on this subject made the comparison either in a non-randomized fashion or were performed in Asia. In the West, most patients with advanced gastric cancer are treated with perioperative FLOT in accordance with current guidelines [23, 25]. Furthermore, minimally invasive surgical procedures are frequently employed for advanced gastric cancer in the West [26, 27]. Meanwhile, in Eastern trials, such an approach was ground for exclusion. Furthermore, the results from Asian studies might not be directly applicable to Western populations due to differences in tumor biology and patient characteristics [35]. The necessity of complete omentectomy in gastrectomy for locally advanced cancer in the Western population is still unclear.

At present, the Japanese gastric cancer guideline recommends a total omentectomy for clinically staged T3–T4 gastric tumors, and partial omentectomy for T1–T2 tumors [24]. Likewise, the National Comprehensive Cancer Network (NCCN) Guidelines advise a total omentectomy during resection with curative intent [36]. Meanwhile, the European Society for Medical Oncology (ESMO) guidelines Tri [23] makes no statement about the necessity of omentectomy in the treatment of gastric cancer, and the Dutch gastric cancer guideline [29] advises to perform at least a partial omentectomy.

In Asia, omentectomy was often performed together with a bursectomy to prevent potential peritoneal metastases. However, a previous trial concluded that bursectomy did not provide a survival benefit over gastrectomy without bursectomy [37]. Therefore, guidelines advise to perform a gastrectomy with D2 lymphadenectomy and omentectomy, without bursectomy, for cT3–4 gastric cancer. Currently, two phase III trials are conducted in Japan [38] and China (NCT04843215). The aim of both trials is to confirm the non-inferiority of omentum preservation compared with omentectomy in patients with cT3 or cT4a gastric cancer in terms of relapse-free survival. However, both trials exclude patients receiving perioperative chemotherapy, and in the Japanese trial patients undergoing minimally invasive gastrectomy are excluded as well. In the current trial, these patients will be included.

Available data indicates that gastric cancer patients might be subjected to futile treatment by performing a total omentectomy; however, high-quality data is lacking in order to confirm the non-inferiority of the omentum-preserving strategy. Furthermore, comprehensive data on the incidence of omental infarction following omental preservation is needed to effectively evaluate the advantages and disadvantages of both strategies. The OMEGA trial will be the first Western randomized controlled trial that will determine if complete omentectomy during gastrectomy for cancer can be omitted in the future.

## Trial status

Protocol Version 6, 12/01/2024. The enrolment of participants began on 01/03/2024. The recruitment is estimated to be completed by 01/03/2026.

## Supplementary Information

Additional file 1: S1. SPIRIT Checklist OMEGA trial. S2. Surgical Quality Assurance assessment form. S3. Surgical Quality Assurance scheme.

Additional file 2

## Data Availability

All data generated during this trial are kept on Castor EDC for 15 years. The datasets will be available from the corresponding author (SSG) upon reasonable request.

## Abbreviations

AJCC: American Joint Committee on Cancer
ASA: American Society of Anaesthesiology
CIPN: Chemotherapy-induced peripheral neuropathy
CRF: Case record form
CRP: C-reactive protein
CT: Computed tomography
D2 lymphadenectomy: Lymph node stations 1–7, 8a, 9, 11p, 11d, 12a
DSMB: Data and Safety Monitoring Board
ESMO: European Society for Medical Oncology
EUS: Endoscopic ultrasound
EQ-5D-5L: Euro Quality of Life-5D-5L
FLOT: Fluorouracil, Leucovorin, Oxaliplatin and Docetaxel
GIST: Gastrointestinal stromal tumor
H&E: Hematoxylin and eosin
HR: Hazard ratio
ICG: Indocyanine green
IQR: Interquartile-range
IRB: Institutional review board
METC: Medical Ethics Review Committee
NCCN: National Comprehensive Cancer Network
NKR: Dutch Cancer Registry
pNET: Pancreatic neuroendocrine tumor
POCOP: Prospective Observational Cohort Study of Oesophageal-gastric cancer Patients
QLQ-C30: Quality of Life Questionnaire C30
QLQ-OG25: Quality of Life Questionnaire Oesophago-Gastric 25
R0: Microscopically complete resection
SQA: Surgical quality assurance
(y) pTNM: (Postneoadjuvant) pathological (p) tumor (T), nodes (N), and metastases (M)
WHO: World Health Organisation
